# Improving oral health service delivery: the patient perspective

**DOI:** 10.1017/S1463423625100637

**Published:** 2025-12-02

**Authors:** Shamiso Chakaipa, Pieter J. Van Dam, Sarah J. Prior

**Affiliations:** 1 Tasmanian School of Medicine, University of Tasmania, Hobart, TAS 7000 Australia; 2 Oral Health Services Tasmania, Hobart, TAS 7008, Australia; 3 School of Nursing, University of Tasmania, Hobart, TAS 7000, Australia; 4 Tasmanian School of Medicine, University of Tasmania, Burnie, TAS 7320 Australia

**Keywords:** patient experience, removable complete dentures, service delivery

## Abstract

**Background:**

Rehabilitation with removable complete dentures (RCDs) involves navigating public dental systems that often present barriers like long wait times and limited access. While clinical outcomes are often known, patient experiences with service delivery remain underexplored. Understanding these experiences is key to improving denture care in public settings.

**Objectives:**

This study aimed to explore and gain a comprehensive understanding of the service delivery experiences of patients rehabilitated with RCDs through the public dental health system in the State of Tasmania in Australia with the goal of informing improvements to the patient journey and overall service quality.

**Methods:**

A qualitative study using a Constructivist Grounded Theory (CGT) approach was undertaken. Twenty-five adult participants who received RCDs between 2017 and 2022 were purposively selected from public dental clinics. Data were collected through in-depth, semi-structured, face-to-face interviews. Analysis followed CGT principles, including iterative coding, constant comparison, memo writing, and the co-construction of meaning with participants.

**Results:**

Participants reported emotional distress linked to prolonged waiting times and limited continuity of care. Despite valuing the professionalism and empathy of individual practitioners, many expressed a need for improved communication, more coordinated interdisciplinary care, and greater system responsiveness. Good service was characterised by accessibility, affordability, approachability, friendly staff, and high-quality care. Suggestions for improvement included moving services close to patients, better integration with other health sectors, and the use of visual aids to support understanding and self-management.

**Conclusions:**

Patient narratives reveal a pressing need to address delays, communication gaps, and fragmented care in the public denture service pathway. System-level changes adopting a more holistic approach such as patient-centred approach, improving interprofessional collaboration, decentralising service provision, and enhancing health communication may significantly improve the denture rehabilitation experience and patient outcomes.

## Introduction

A core determinant of healthcare organisations’ success is the quality of patient experience. Positive patient experiences foster trust, improve engagement, and enhance service delivery, ultimately contributing to better health outcomes (Australian Commission on Safety and Quality in Health Care, 2021; Gleeson *et al.*, 2016). Both patient satisfaction and experience are essential metrics, capturing not only clinical outcomes but also patients’ perceptions of their care journey. Many healthcare providers use patient satisfaction feedback to gain insight into their services and identify areas for improvement (Manzoor *et al.*, 2019; Ferreira *et al.*, 2023). While satisfaction is a key quality indicator, patient experience goes further by evaluating the responsiveness, effectiveness, and person-centredness of care delivery (Swathi *et al.*, 2023). It encompasses dimensions such as communication, emotional support, accessibility, continuity, and respect for individual preferences (Manzoor *et al.*, 2019; Ferreira *et al.*, 2023). In complex systems where care is often fragmented, understanding lived experiences enables providers to address service gaps, enhance trust, and tailor care to meet patient needs, ultimately supporting a more holistic and person-centred approach (Manzoor *et al.*, 2019; Ferreira *et al.*, 2023).

With the shift towards more holistic models, patient experience now lies at the heart of patient-centred care (PCC). PCC is defined as care that is respectful of and responsive to individual preferences, needs, and values, ensuring that these values shape all clinical decisions (Institute of Medicine, 2001; Araki, 2019). The progression from measuring satisfaction to understanding experience marks a commitment to viewing patients as active participants rather than passive recipients of care. PCC promotes empowerment by encouraging patients to share feedback and play a role in shaping their care journey (Wong *et al.*, 2020). It also fosters collaboration between patients, families, and providers, which strengthens trust, supports adherence, and leads to improved satisfaction and health outcomes (Wong *et al.*, 2020). Furthermore, engaging patients enhances provider efficiency by streamlining communication and reducing unnecessary interventions, allowing for more targeted care (Marzban *et al.*, 2022; Chakaipa *et al*., 2023).

In Australia, Standard Two of the National Safety and Quality Health Service Standards ‘Partnering with Consumers’ emphasises the critical role of collaboration in healthcare (National Safety and Quality Health Service, 2017). This standard promotes meaningful partnerships where patients are empowered to be co-creators of their healthcare experience. Research shows that such collaborations improve service planning, communication, and even provider attitudes, contributing to safer, higher-quality care (Torres *et al.*, 2019). These partnerships have also been linked to reduced hospital costs, shorter lengths of stay, and better outcomes when based on shared decision-making and mutual respect (Bombard *et al.*, 2018; Maher *et al.*, 2017).

Patient feedback plays an instrumental role in enhancing the quality of PCC in public hospitals (Wong *et al.*, 2020). Tools such as Patient Reported Experience Measures (PREMs) and Patient Reported Outcome Measures (PROMs) are increasingly embedded in routine practice (Kumah *et al.*, 2017; Chakaipa *et al*., 2023). PREMs assess aspects like communication, respect, accessibility, and decision-making involvement (Australian Commission on Safety and Quality in Health Care, 2021) while PROMs focus on health status, functioning, symptoms, and quality of life before and after care (Kumah *et al.*, 2017; Chakaipa *et al*., 2023). Collecting and acting on this feedback not only improves services, but also boosts employee satisfaction and reduces staff turnover, an urgent concern amid ongoing workforce shortages (KPMG, 2024). Incorporating patient feedback into quality improvement initiatives ensures care is more aligned with patient needs, resulting in sustainable improvements (Cadel *et al.*, 2022). Such care includes oral health which services, spans preventive to rehabilitative care, delivered across diverse settings including clinics, hospitals, and private practices (Australian Commission on Safety and Quality in Health Care, 2015). Incorporating patient experiences into oral health policy and practice can significantly improve service alignment and health outcomes. PROMs in dentistry are increasingly used to ensure that treatments are aligned with patient values and expectations (Leonardsen *et al.*, 2020; Chakaipa *et al*., 2023; Friedel *et al.*, 2023) which differs from traditionally underutilised patient experience data, focusing instead on minor service adjustments without addressing deeper clinical behaviours (Price *et al.*, 2014). Additionally, engaging patients meaningfully in the design and implementation of oral health services has been shown to improve patient experiences (Chakaipa *et al*., 2023). Exploring and acknowledging the myriad of historical challenges that exist for oral health patients and utilising these experiences to support change will ensure person-centred improvements are designed and implemented (Chakaipa *et al*., 2023). Historically, oral health patient experience data has primarily been collected through quality-of-life surveys and used to identify minor service improvements, such as simplifying admission processes or providing educational materials, with little impact on clinician behaviour (Price *et al.*, 2014). A gap exists in oral health research that necessitates the use of more robust methods to capture patient experiences for improving service delivery using qualitative studies. Therefore, the aim of this study is to explore how patients seeking removable complete denture (RCD) rehabilitation experienced and navigated oral health care, to inform improvements in service delivery.

## Methods

This study employed a qualitative approach using an iterative process for data collection and analysis (Charmaz, 2006). The Constructivist Grounded Theory (CGT) methodology, as developed by Charmaz (2006) was selected to develop a comprehensive and contextualised understanding of the service delivery experiences of patients rehabilitated with RCDs. CGT allows for the investigation of understudied phenomena like patient experiences in an oral public health. Moreover, GCT recognises that knowledge is co-constructed between researcher and participants, making it appropriate for understanding the complex, subjective nature of service delivery experience. Its iterative and reflexive processes enable concepts to emerge from participants’ realities, ensuring that the findings capture patient perspectives while offering insights to improve responsive and PCC.

### Sampling strategy

Purposive sampling was used to recruit adults who received complete dentures via public oral health services in Tasmannia between 2017 and 2022. The criteria for sampling are outlined in Table 1.

**Table 1.** Purposive sampling criteria

Inclusion	Exclusion
Removable complete denture upper and lower	Upper and lower dentures partial dentures
Removable complete upper denture opposing lower dentate	Less than 1 month of denture wearing
Removable complete upper denture with no opposing lower	More than 5 years of denture wearing
Removable complete lower denture with opposing dentate	
Received treatment within OHST 5 years or less	
Ability to understand English language	

### Data collection

Data were collected through face-to-face semi-structured interviews, audio-recorded, and transcribed verbatim. Interview questions, informed by previous research (Chakaipa *et al*., 2023), explored participants’ denture rehabilitation experiences and interactions with public oral health services. As data collection progressed, theoretical sampling guided refinement of interview questions to explore emerging themes. Final interviews tested overarching concepts identified earlier in the data collection stage.

### Data analysis

Data were analysed using the iterative principles of CGT (Charmaz, 2006), progressing through initial coding, focused coding, and theoretical sampling. Initial coding was conducted line by line to stay close to participants’ language. For example, a statement such as ‘I avoided eating in public because I felt ashamed’ was coded as ‘withdrawing from social eating due to shame’. During focused coding, recurring initial codes were synthesised into more conceptual categories, such as ‘managing social stigma’ and ‘reframing self-image.’ As categories developed, theoretical sampling was used to recruit participants who could deepen or challenge emerging concepts – for example, those with diverse dietary practices to refine the category ‘adjusting to dietary changes’.

NVivo 12 supported systematic data management, allowing for consistent coding, hierarchical organisation of categories, and memo writing. It also facilitated transparency and comparison across interviews, enhancing analytical rigour and the development of the substantive theory. Methodological rigour was maintained through several strategies aligned with qualitative research standards (Lincoln and Guba, 1985). **Constant comparative analysis** ensured analytical depth and consistency across interviews. **Participant validation** was employed during follow-up conversations, allowing participants to confirm or refine emerging interpretations, thus enhancing **credibility**. Although coding was primarily conducted by one researcher, **peer debriefing** sessions with experienced qualitative scholars supported reflexivity and acted as a check for **dependability**. **Analytic memos** tracked theoretical development and decision-making throughout the process. The use of NVivo 12 further supported rigour by enabling systematic coding, transparent data management, and an auditable analytic trail. Together, these strategies strengthened the **trustworthiness** of the findings.

## Results

Twenty-five interviews were completed, and Table 2 presents the participant characteristics. A diverse sample of 25 participants was included, reflecting varied socioeconomic and geographic backgrounds to capture a broad range of experiences. The sample size was determined by data sufficiency, where recruitment continued until the data collected were adequate to fully develop categories and explain the phenomena under study. At this point, additional interviews no longer contributed to new insights, indicating that the sample was sufficient to capture the complexity and diversity of patient experiences.

**Table 2.** Demographic data for participants

Phase	Number of participants	Age range (years)	Male (*n*)	Female (*n*)
Phase 1 (Initial interviews)	6	50–70	3	3
Phase 2 (Theoretical sampling)	4	60–75	2	2
Phase 3 (Further theoretical sampling)	4	50–75	2	2
Phase 4 (Testing theoretical concepts)	11	45–70	8	3
Total	25	45–75	15	10

Patients’ experiences of service delivery during their rehabilitation with RCDs took place from the commencement of tooth loss to living with RCDs after the rehabilitation process. This includes four categories of service delivery experience which are defined in Table 3. Each of these categories is then described in full with evidence from participants to support the findings.

**Table 3.** Service delivery categories and their descriptions

Experience categories	Description
Responding to the waitlist	This category captures participants’ realisation that accessing public oral health services involved being placed on a waitlist, delaying the start of their removable complete denture rehabilitation. It highlights their responses to the extended waiting period before treatment began.
Responding to expectation of service	This category highlights the contrast between participants’ initial expectations of service delivery and the care they actually received.
	It also reflects their recognition of the medical care as respectful, attentive, and responsive to their individual experiences, preferences, needs, and values.
	Provides an understanding of how participants advocated for written instructions, such as post-insertion care and cleaning tips to include images in pamphlets as more effective than verbal explanations.
	Captures how participants provided an evaluation of their experiences based on what they personally considered to be quality or satisfactory care.
Bringing others on board.	Reflect the extent to which the cooperation between dental professionals and other non-dental healthcare providers benefitted participants, emphasising the positive influence of this interdisciplinary collaboration on the quality, continuity, and overall experience of their care.
	Reveals cooperative partnership between the patient and their healthcare provider that enables the patient to actively participate in their treatment, allowing them to initiate discussions, offer suggestions, and help shape the direction of their care.
Moving and integrating services closer	Reflects on participants’ desire for oral health services to be offered within their local communities and located near other healthcare facilities for greater convenience and accessibility

### Category 1: responding to the waitlist

All patients requiring oral health services are placed on a waiting list for appointments according to their clinical needs and urgency. Although participants expressed that they understood that waiting was a common part of public healthcare, the length of the waitlist came as a surprise when they were informed.

*Initially, when I went to Oral Health Services, right from the beginning, I had to wait two years or something like that.* P8

Participants reported that their experiences of the oral health rehabilitation journey on the waitlist were emotionally challenging, requiring resilience, and adaptability. Many described struggling to manage their expectations and cope with the uncertainty of the process of waitlist. This led to a mix of emotions, frustration at the delays, acceptance of the system’s limitations, and determination to persevere through the waiting process. Participants shared the struggles they faced while on the waitlist, highlighting the toll that the extended waiting period took on their oral health. Many expressed concerns that their conditions were worsening the longer they remained on the list, with some even experiencing secondary problems as a result. As their conditions deteriorated, participants often found themselves facing more complex and costly treatments, which could have been avoided with an earlier intervention. This sense of escalating discomfort and the fear of further complications created additional stress and frustration. For many, the waitlist was not just a matter of time –it was a constant source of anxiety as they watched their conditions deteriorate, leading to a cycle of increasing pain and medical complexity. Participants asked to be moved up the waitlist, but often this did not ease the anxiety and pain associated with waiting.

*And I explained to her that, look, I was getting sicker and that, could this be escalated, or could I be moved up the list by any chance? Which they’d done.* P14

Some participants acknowledged the complexities of public healthcare systems while maintaining hope for improvement. For these participants, the waitlist was not merely a barrier but a reflection of larger societal priorities, prompting discussions about the need for better investment in oral health to ensure equitable access for all. They called for improvements in turnaround times, better communication about expected timelines, and possibly increased workforce capacity or resource allocation to speed up the process.

*I’m, I mean I’m not an expert by any means, but I’m just trying to think of what budgetary constraints there would be, as well as where the need would be greatest.* P10

*Obviously, once again, if there’s more funding, that (waiting) period of time would be reduced drastically, yes.* P8

*If there were more possible surgeons available and places, and it’s not just the dental service, it’s also what the hospital will allow, as well, for the surgeons to be able to do their procedures.* P14

Another reported challenge was the lack of communication and feedback regarding their position on the waitlist. Many expressed frustrations over not knowing when they could expect treatment. This uncertainty added to the emotional burden of waiting, as participants were left in the dark about their progress and had little clarity on how long the wait would last. The call for better transparency and more frequent updates became a key suggestion for improving the patient experience, helping to reduce anxiety and build trust in the system.

*Why won’t they update me (on waitlist progression) to get (my teeth) fixed.* P1

Upon commencing rehabilitation, participants discovered they were placed on another internal waitlist with lengthy turnaround times. Many described how delays between appointments and inefficient scheduling prolonged their treatment, causing ongoing discomfort and uncertainty. For these individuals, the wait was not just inconvenient, but it impacted their daily lives significantly. Participants described that without teeth, they faced both practical challenges in eating and speaking, and emotional distress from feeling incomplete, highlighting the physical and psychological toll of prolonged temporary edentulism during rehabilitation.

*And I didn’t know that I was going to go through three months (of waiting without any teeth) of speech impediments of difficulty eating, a change of habits and stuff like that, to the extent that I had to experience.* P1

*That I’m going to be toothless for, like, six months. It did. It made me conscious, straight up, that it’s like, oh, hang on, do I really want to do this? Do I want to really go through the next six months without having any teeth in my head?* P15

### Category 2: responding to expectation of service

Participants discussed how the service they received matched their expectations of the service. While some participants shared a deep appreciation, recognising it as a vital aspect of their overall treatment experience, others did not find this to be the case. Many participants highlighted how practitioners took time to understand their unique concerns and circumstances, tailoring the treatment approach to meet their specific needs. This personalised care extended beyond clinical procedures, as practitioners facilitated ongoing interactions and ensured that patient’s interests were addressed throughout the therapy process. For these participants, the attention to detail and individualised care created and made them to feel that they were in a supportive environment that enhanced their overall satisfaction and sense of well-being. They confirmed that they experienced empathy and were effectively communicated to, some through check ins during appointments.

*And she kept asking through that process, are you okay, do you need a break?* P14

Other participants who expressed overall satisfaction with their oral health services noted consistent, reliable, and predictable care, which helped build trust and reduce uncertainty. However, some felt communication could be improved, particularly regarding appointment details or visit clarity. Others acknowledged that the service’s capacity might be impacted by funding constraints and differing viewpoints. Despite these concerns, those satisfied with the service valued its availability when needed. Key concepts within this category included the advantages of public oral health, personalised care, defining a good service, and the importance of having competent, honest, respectful, and friendly practitioners. The majority of participants perceived public oral health services as beneficial and aligned with their expectations, with the quality of overall service, not just direct care, playing a significant role in shaping their experiences. In particular, feeling at ease and welcomed by staff was appealing to the overall process.

*But the people that work there, you people in the back, you always come out to get the patient. That’s a very nice gesture. (You know) It’s the doctor will see you now, and when you walk out, and you call (me by) my name. That’s personalised service.* P12

*I feel very welcome here (public oral health services Tasmania), and I feel like it’s a place of safety and sanctuary. It made a big difference.* P7

For others, positive experiences with practitioners were defined by the respectful and compassionate nature of the interactions. They described their practitioners as friendly, truthful, and competent, feeling that they were treated as equals rather than as inferior. This approach fostered a sense of dignity and trust, allowing participants to feel comfortable and valued throughout their treatment journey. These participants appreciated that their practitioners took the time to explain procedures, answer questions, and involve them in decision-making, creating a collaborative environment where their concerns were heard and addressed. The respectful demeanour and professionalism of the practitioners helped reduce any anxiety or discomfort participants might have felt, making the overall experience more positive.

*Whereas I walk into the dental office, and I walk in there knowing that I’m going to be treated like a person, and that people care. And that’s a big factor. I’m not a number. I’m a real person.* P11

*She was always nice and friendly and talkative and didn’t treat you like you were inferior.* P13

For these participants, the relationship with their practitioners went beyond clinical care, it was about mutual respect, understanding, and competence, which played a key role in enhancing their confidence and satisfaction with the treatment they received. Some participants highlighted that they received good service and characterised good service as being accessible, affordable, approachable, and available. They viewed good service as meaning that it has friendly, accommodating, kind, sound advice offering, and quality clinical care staff. The environment should be clean, with well-designed waiting areas and well-maintained surroundings, and staff should be pleasant with effective communication skills.

*Well, a good service to me looks like it’s very approachable. It’s approachable as in, welcoming as well.* P7

*I think I like the fact that you can ring up at eight o’clock and see somebody today if your story’s good enough, and I think that’s fantastic. If you could do more of it, it would be better.* P9

*Accessible and affordable (is what a good service means). I think I haven’t had a problem with (Oral Health Service Tasmania).* P10

Some participants reported poor service experiences and feelings of being mistreated. They described communication breakdowns and a lack of prioritisation, such as waiting an entire day at the clinic only to be told to return and wait again the next day. Some felt dehumanised during treatment, where initial interactions seemed personal but quickly became mechanical, reducing them to procedures rather than individuals.

*So, it’s only the first ten minutes that I’m actually me, or I’m me. And then after that it’s like, now sit down, do this, open your mouth, spit. It’s all about you guys, and what you’re doing. I don’t think it’s about me as a man. I think it’s about your teeth, that’s it. But they’re putting in teeth, they should worry about the person they’re putting the teeth in.* P20

Participants felt the focus was solely on their teeth, with little attention to their overall well-being, and complications were often met with frustration rather than empathy or support.

*So, what I was dealing with, I didn’t realise I had dry sockets. And he was pissed. He was not happy. He was really angry with me.* P23

Some participants reported limitations in communication and suggested improvements in communication were needed to understand the before and after care particularly using pamphlets and images. They emphasised the importance of clear, visual aids for post-treatment care and self-management. Written instructions, such as post-insertion care and cleaning tips, were considered more effective than verbal explanations, as they provided a tangible reference. Participants who recommended providing prior and post-insertion instructions in pamphlet form believed this will ensure consistency and accessibility, especially for older individuals who may struggle with memory.

*So, basic instructions, not that people will probably do it anyway, but I’m just saying that basic instructions are necessary to be given.* P10

I *think a little bit of information would be helpful to other patients, knowing something that’s easy to read for older patients, because I know a lot of people are older. And just little tips like that to help with cleaning the denture with.* P14

### Category 3: bringing others on board

Participants acknowledged existing interdisciplinary collaboration in their care but felt it could be expanded. They recommended involving professionals such as Psychologists, Counsellors, General practitioners, and Pharmacists to improve the overall treatment experience. Participants recognised that oral health concerns often overlap with broader health and emotional issues, including pain and the psychological effects of tooth loss. They believed a more integrated approach would offer holistic care by addressing both physical needs and emotional well-being. It was suggested that including mental health professionals could create safe, supportive spaces for patients facing anxiety, distress, or frustration during treatment needs that oral health practitioners alone may not be equipped to fully manage.

*…, I think if there was a psychologist or a counsellor where someone had a safe space to talk to people about the teeth issue, or their anxiety, or the fact that it’s taking so long to get through the process.* P14

Others suggested that psychiatrists could play a role in managing more complex psychological conditions, especially when working in collaboration with dentists to ensure comprehensive care.

*A psychiatrist (will be useful) if a person is having any problem. (Working) In conjunction with all the dentists and all that, would be great.* P11

Pain management was another concern, with participants noting the role of pharmacists or chemists in providing temporary relief through appropriate medications before dental appointments. The inclusion of chiropractors was acknowledged in managing jaw-related pain, particularly when denture wear or oral procedures result in joint or muscular discomfort by other participants.

*I think pain management’s a little underdone, definitely, as a prelude to visiting you guys. And it would be really nice if the chemist could give you enough of whatever you guys think is good tooth drugs, and just give someone three, four days of that.* P9

*The chiropractor fixed XXX jaw when she had some pain. You’re bound to have come across where it was a little bit displaced. And he fixed that just by, it took a few goes.* P9

Participants valued partnering with practitioners during rehabilitation, which empowered them to take an active role in their care. This collaborative approach enabled them to express preferences, make decisions about treatment timing and nature, and feel heard by their providers. They felt that practitioners supported this process by offering expert advice and guiding informed decision-making. As a result, participants reported greater satisfaction and a stronger sense of responsibility for their treatment outcomes. Being involved in decision-making gave them a feeling of control over their rehabilitation journey, reinforcing the importance of shared responsibility and mutual respect in achieving positive oral health outcomes.

*But the bottom plate, I now researched a little more carefully, and I suggested (to the practitioner) is there any way you can save? He said those two bicuspids at the bottom, there’s three of them, they seem to be all right. But they weren’t any different to the rest of them, so they pulled the rest and left those as an anchor for my lower plate. And they’re still there today and that’s 20 years.* P12

### Category 4: moving and integrating services closer

Some participants emphasised the importance of improving access to dental care by moving services closer to where people live, particularly in remote or suburban areas. They believed that bringing services geographically closer would reduce travel burdens, long commute times, and traffic congestion, making it easier for patients to attend appointments and receive timely care. Additionally, participants highlighted the benefits of service integration, suggesting that better coordination among healthcare providers, including referral systems, could streamline care. This would reduce the need for patients to travel between locations for different aspects of treatment. Integration was seen to improve the continuity and convenience of care by minimising multiple appointments across various facilities.

*And if you’ve got a focus where the need is greatest, then your need is probably going to be in those poorer areas, like New Norfolk as an example. So, if you had one at Bridgewater, Risdon Vale, Claredon Vale and Rokeby and down through there.* P10

Overall, moving and integrating services were viewed as practical strategies to enhance accessibility, ensure equitable service delivery, and reduce barriers to care particularly for those in underserved or rural communities leading to a more efficient and patient-centred oral healthcare experience.

## Discussion

This study examined the experiences of patients receiving public oral health services in Tasmania focusing on opportunities for service improvement. It identified four key categories: responding to the waitlist, responding to service expectations, bringing others on board, and moving services closer. Findings revealed how participants navigated delays, assessed care quality, valued collaborative interdisciplinary support, and appreciated localised service access. The study highlighted structural and relational factors shaping experiences during complete denture rehabilitation, showing how patients connected their personal journeys with wider system challenges and the importance of socially and clinically responsive care environments in enhancing overall service delivery and satisfaction.

One of the most significant findings was the burden imposed by lengthy waitlists. Participants frequently described waitlists as excessively long, corroborating previous research by Dudko (2019) and Lalloo and Kroon (2015) which found that dental waitlists continued to grow across many states in Australia. Our study has provided new insights into patients’ experiences placed on an oral health waitlist. The burden of oral health waitlists has not been investigated previously in detail, particularly concerns regarding the impact of waitlist on oral patient experiences and outcomes appears to be under reported. However, a study conducted in Finland (Tuominen and Eriksson, 2012) investigating patient experiences during waiting for dental treatment found that a majority of patients reported low levels of inconvenience, but the level of inconvenience increased after a period of four months. This is consistent with the findings of our study, where patient expressed concerns that their conditions were worsening the longer they remained on the waitlist. Our study found that anxiety levels increased while being on the waitlist. Increased anxiety levels, depression and reduce quality of life related to waitlists has been reported for conditions like organ transplant, surgery and cancer management. (Gagliardi *et al.*, 2021). The same study (Gagliardi *et al.*, 2021) also found that patients concerns should be acknowledged and that regular communication about the waitlist position should be a priority. The lack of communication in our study was regarded as a major issue. Moreover, the implications of delays in treatment include minor oral health issues often progressing into more complex conditions or even resulting in tooth loss (Dudko, 2019).

Our findings reinforce the view that long waitlists are a structural component of public healthcare in Australia (Dudko *et al.*, 2016; Lalloo and Kroon, 2017) which undermine preventative care and exacerbate oral health disparities, thus efforts need to be strengthened to tackle waitlists and assist to bring them down especially in oral health settings. However, studies show that such attempts have been explored before through the Australian National Partnership Agreements (NPAs) on public dental services of 2012 which temporarily alleviated dental wait times through capped subsidised private care (The Australian Government Department of Health, 2012). Despite such measures being taken, it appears that the efforts have been insufficient, leading to cumulative wait times over the years. Current efforts to address waitlists remain fragmented, highlighting the need for a comprehensive and integrated oral health policy framework. Moreover, a PCC policy framework should take into consideration the complexities of oral health and it should ensure that the right care, in the right place at the right time, tailored to the needs of the community is clearly stated (Maree *et al.*, 2020).

The use of private providers remains an underutilised strategy. One of the solutions put forward by Duckett *et al.* (2019) is a universal dental scheme, whereby publicly funded dental care should be delivered by a mix of public and private providers. Shifting funding arrangements could lead to a holistic approach in providing oral health care and treatment.

Most participants in this study largely described their interactions with dental practitioners in favourable terms, articulating friendliness, respect, competence, and honesty. However, others revealed the opposite including breakdown in communication, dehumanisation and lack of practitioner support, empathy and sympathy. Such findings align with previous research by Liang and Howard (2023) showing that most patients generally report positive clinician interactions over negative. However, the same study argued that even a small proportion of negative encounters if not addressed can affect trust and treatment adherence.

The idea of personalised care emerged within this study with participants showing appreciation for treatment that was tailored to their individual needs and values, including clear and compassionate communication. These findings align with the concept and synthesis of PCC models by Wasim *et al.* (2023), where rapport and relationship-building were foundational components. Thus, our study solidifies our earlier proposal that effective denture rehabilitation should extend beyond technical competence to include clear, empathetic, and compassionate communication, as well as recognition of the person behind the patient creating a relational approach to care where *how* treatment is delivered becomes just as important as *what* treatment is delivered. This concept would be even stronger if interdisciplinary collaboration was seen as the norm, whereby a holistic form of care coordination and sharing of data becomes the essence of the service provided (Böhme Kristensen *et al.*, 2023).

Improvements in communication was suggested by providing pamphlets illustrating treatment stages, which participants suggested would be helpful in self-managing care further reinforcing Wasim *et al.*’s (2023) emphasis on communication as central to effective PCC. This study revealed the need to increase existing efforts on interdisciplinary collaboration in oral health care with participants expressing a need for the inclusion of professionals beyond dentistry. Thus, interdisciplinary collaboration extending to psychologists, dieticians, physiotherapists, and counsellors to support their rehabilitation process holistically is central. The potential benefits of such models are well-documented in other fields (Slatsveen *et al.*, 2023; Warren and Warren, 2023) and their application in oral health could significantly enhance patient outcomes.

Moving and integrating services closer featured as another key theme. Participants advocated for services to be made available closer to their residential areas, citing challenges related to mobility and transportation, particularly for older adults with comorbidities. This reinforces the view that decentralised services enhance access and equity (McIntyre and Klugman, 2003; Rotulo *et al.*, 2023).

While substantial progress has been made in child dental services in Tasmania similar investments are needed for adult (Auditor General Report, 2024). A phased moving of services to outer suburbs strategy could yield significant benefits, particularly for vulnerable populations such as those undergoing complete denture rehabilitation (Rushton *et al.*, 2021; Sapkota *et al.*, 2023).

Finally, the quality of the practitioner-patient relationship was instrumental in shaping patient experiences. Participants frequently described how strong rapport facilitated open conversations, emotional support, and greater treatment acceptance. This study demonstrates that empathetic communication made participants feel understood and respected, echoing findings from Wasim *et al.* (2023) and Zavanelli *et al.* (2017) who underscore empathy as a cornerstone of therapeutic relationships.

### Study contribution

This study enhances understanding of patient experience by exploring how individuals engage with public oral health services during denture rehabilitation. It reveals key issues like system gaps, practitioner relationships, interdisciplinary needs, and service access providing insights that can inform better clinical and policy decisions.

### Practical and clinical implications

Findings highlight the importance of strengthening PCC and improving service coordination. Clinicians should support active patient involvement, while policymakers need to address fragmented waitlist systems, invest in regional service delivery, and promote interdisciplinary collaboration to boost outcomes and satisfaction.

### Limitations

As the study focused on a single regional Southern Tasmanian site with English-speaking, self-selected participants, findings may not be widely generalisable. However, the rich accounts offer meaningful insights into the public oral healthcare experience of denture wearers.

### Directions for future research

Further research could explore experiences of non-English-speaking populations, metropolitan and interstate settings and assess how decentralised, team-based models affect care access and satisfaction over time.

### Conclusion

The matter of oral health service delivery issues of patients rehabilitated with RCDs is an under investigate area of research. This study has contributed to the body of knowledge, exploring the patient experience, providing a comprehensive understanding of service delivery concerns. The findings of this study emphasised that the denture rehabilitation journey is emotionally and practically challenging due to long wait times, unclear communication, and difficulties with daily functioning. Nevertheless, participants valued the respectful, empathetic care received during treatment and appreciated being involved in decision-making. They called for more accessible, integrated services particularly for rural and suburban communities and highlighted the importance of supportive, collaborative practitioner relationships. The study reinforces the need for empathetic, person-centred, and well-coordinated public oral health services and this should be reflected in a comprehensive and integrated oral health policy framework addressing access to oral health and equity. The development of clear strategies for the use of private providers to reduce waitlists, interdisciplinary collaboration, and the development and implementation of staff education for better communication should be considered.

## Data Availability

Data access not made available due to strict privacy organisational policy.
